# A Nationwide Genomic Study of Clinical Klebsiella pneumoniae Carrying *bla*_OXA-232_ and *rmtF* in China

**DOI:** 10.1128/spectrum.03863-22

**Published:** 2023-04-27

**Authors:** Siquan Shen, Renru Han, Dandan Yin, Bo Jiang, Li Ding, Yan Guo, Shi Wu, Chuning Wang, Hong Zhang, Fupin Hu

**Affiliations:** a Institute of Antibiotics, Huashan Hospital, Fudan University, Shanghai, China; b Key Laboratory of Clinical Pharmacology of Antibiotics, Ministry of Health, Shanghai, China; c Department of Clinical Laboratory, First Affiliated Hospital of Kunming Medical University, Kunming, China; d Department of Clinical Laboratory, Shanghai Children’s Hospital, Shanghai Jiaotong University, Shanghai, China; Institut Pasteur

**Keywords:** *Klebsiella pneumoniae*, *bla*_OXA-232_, *rmtF*, genetic context, genetic environment

## Abstract

OXA-232 carbapenemase is becoming a threat in China due to its high prevalence, mortality, and limited treatment options. However, little information is available on the impact of OXA-232-producing Klebsiella pneumoniae in China. This study aims to characterize the clonal relationships, the genetic mechanisms of resistance, and the virulence of OXA-232-producing K. pneumoniae isolates in China. We collected 81 OXA-232-producing K. pneumoniae clinical isolates from 2017 to 2021. Antimicrobial susceptibility testing was performed using the broth microdilution method. Capsular types, multilocus sequence types, virulence genes, antimicrobial resistance (AMR) determinants, plasmid replicon types, and single-nucleotide polymorphism (SNP) phylogeny were inferred from whole-genome sequences. OXA-232-producing K. pneumoniae strains were resistant to most antimicrobial agents. These isolates showed partial differences in susceptibility to carbapenems: all strains were resistant to ertapenem, while the resistance rates to imipenem and meropenem were 67.9% and 97.5%, respectively. Sequencing and capsular diversity analysis of the 81 K. pneumoniae isolates revealed 3 sequence types (ST15, ST231, and one novel ST [ST-V]), 2 K-locus types (KL112 and KL51), and 2 O-locus types (O2V1 and O2V2). The predominant plasmid replicon types associated with the OXA-232 and *rmtF* genes were ColKP3 (100%) and IncFIB-like (100%). Our study summarized the genetic characteristics of OXA-232-producing K. pneumoniae circulating in China. The results demonstrate the practical applicability of genomic surveillance and its utility in providing methods to prevent transmission. It alerts us to the urgent need for longitudinal surveillance of these transmissible lineages.

**IMPORTANCE** In recent years, the detection rate of carbapenem-resistant K. pneumoniae has increased and represents a major threat to clinical anti-infective therapy. Compared with KPC-type carbapenemases and NDM-type metallo-β-lactamases, OXA-48 family carbapenemases are another important resistance mechanism mediating bacterial resistance to carbapenems. In this study, we investigated the molecular characteristics of OXA-232 carbapenemase-producing K. pneumoniae isolated from several hospitals to clarify the epidemiological dissemination characteristics of such drug-resistant strains in China.

## INTRODUCTION

The widespread prevalence of carbapenem-resistant *Enterobacterales* (CRE), with the characteristic of extensive drug resistance, leads to high mortality and poses severe challenges to clinical infection management (1). The increase of CRE is mainly due to the emergence and spread of carbapenemases, mainly including KPC, NDM, IMP, VIM, and OXA-48-like enzymes (2). Although KPC and NDM are the most widespread carbapenemases in China, the OXA-48-like lactamases have recently been reported successively in different regions of China, which cannot be ignored (3). To date, many OXA-48-related variants have been reported, including OXA-232, OXA-181, OXA-163, etc. (4). As one of the most common global OXA-48-like derivatives worldwide, OXA-232 was first described in 2013 in France from one Klebsiella pneumoniae isolate and two Escherichia coli isolates. Since then, OXA-232-producing K. pneumoniae isolates (OXA-232-Kp) with the sequence types (STs) ST14, ST15, ST16, ST17, ST147, ST231, ST307, and ST395 have been reported worldwide (5–14). Compared to OXA-48, OXA-232 showed low ability to hydrolyze carbapenems (15). Almost all OXA-232-Kp isolates had plasmids with more than 99% sequence identity to pKNICU5, a ColKP3-type plasmid with OXA-232 in a truncated Tn*2013* (7, 15). The genetic context surrounding *bla*_OXA-232_ is very similar to that surrounding *bla*_OXA-181_ in plasmid pKP3-A, except for a large deletion at the 5′ end of the IS*Ecp1* transposase gene in pOXA-232 (15, 16).

Aminoglycosides are used for severe infections caused by Gram-negative organisms and have shown reliable efficacy in combination with carbapenems, which can effectively reduce mortality (17). The 16S rRNA methyltransferase (16SRMTase) genes, commonly found in *Enterobacterales* worldwide, confer high levels of resistance to aminoglycosides, including *armA*, *npmA*, and *rmtA* through *rmtH*. The most common 16SRMTase-encoding gene in China is *rmtB*, followed by *armA* (18). While *rmtF* was first identified in France in 2012 (19), it has subsequently been reported sporadically in the United States, India (20), the United Kingdom (20), China (21), and other regions successively (12). In recent years, relevant reports of *rmtF* have emerged, always accompanied by harboring of *bla*_OXA-232_ (9, 22).

In the previous studies, *bla*_OXA-232_ is mainly associated with nosocomial outbreaks in many countries, including China (14, 22), the United States (23), South Korea (24), and Brunei (9). Here, a comprehensive overview of the epidemiology of 81 OXA-232-Kp isolates in China was completed to identify the genomic characterization of *bla*_OXA-232_ and *rmtF*, including dominant STs, clonal distribution, and virulence, and with a particular focus on the emergence and dissemination of extended-spectrum β-lactamases (ESBLs), AmpC, and outer membrane porins (Omp).

## RESULTS

### Characteristics of the OXA-232-Kp isolates studied in this study.

The collection consisted of 81 isolates from patients ranging in age from 2 days to 91 years, as shown in Table 1. The most common departments were the neonatal intensive care unit (61.7%), intensive care unit (12.3%), general surgery department (7.4%), respiratory medicine department (3.7%), neurosurgery department (1.2%), and other departments (13.6%). Most isolates were obtained from sputum (60.5%) and broncho-alveolar lavage (18.5%). Antimicrobial susceptibility testing revealed that all 81 isolates were resistant to cefepime, ceftolozane-tazobactam, amikacin, ceftazidime, aztreonam, levofloxacin, ciprofloxacin, piperacillin-tazobactam, and trimethoprim-sulfamethoxazole, with most being resistant to cefepime-tazobactam (1.2% susceptibility). Most of them were susceptible to ceftazidime-avibactam (100% susceptibility), tigecycline (98.8% susceptibility), cefepime-zidebactam (93.8% susceptibility), and polymyxin B (98.8% susceptibility). However, these isolates showed partial differences in susceptibility to carbapenems: all strains were resistant to ertapenem, whereas the resistance rates to imipenem and meropenem were 69.1% and 98.8%, respectively (Table 2).

**TABLE 1:** Age distribution of 81 OXA-232-Kp isolates from patients

Age (yrs)	Percent (%) of isolates
<1	76.50%
11–20	3.70%
30–40	1.20%
40–50	7.40%
50–60	6.20%
70–80	1.20%
80–90	2.50%
>90	1.20%

**TABLE 2:** Antimicrobial susceptibility profiles of OXA-232-KP^*a*^

Strain	ST	Carbapenemase	MIC (mg/L) for:																	
			ETP	IPM	MEM	FPZ	FPT	CZT	CZA	FEP	TGC	AMK	POL	CAZ	ATM	CIP	LVX	TZP	SXT	CRO
1588	231	OXA-232	64	32	32	1	64	128	1	>128	2	>128	0.5	>32	>128	>8	>16	>256	>32	>32
1590	231	OXA-232	128	32	32	1	64	128	1	>128	2	>128	0.5	>32	>128	>8	>16	>256	>32	>32
1596	231	OXA-232	16	16	32	1	64	128	1	128	2	>128	0.5	>32	>128	>8	>16	>256	>32	>32
1601	231	OXA-232	64	8	32	2	64	128	2	>128	2	>128	1	>32	>128	>8	>16	>256	>32	>32
1616	231	OXA-232	8	32	32	1	64	128	1	>128	2	>128	0.5	>32	>128	>8	>16	>256	>32	>32
1617	231	OXA-232	16	32	32	1	>64	>128	1	>128	2	>128	0.5	>32	>128	>8	>16	>256	>32	>32
1847	15	OXA-232	32	8	16	0.25	16	32	0.25	128	1	>128	0.5	32	128	>8	>16	>256	32	>32
KM1	231	OXA-232	128	4	32	1	64	>128	1	>128	1	>128	1	>32	>128	>8	>16	>256	>32	>32
KM2	231	OXA-232	>128	16	64	1	>64	>128	1	>128	2	>128	0.5	>32	>128	>8	>16	>256	>32	>32
KM3	231	OXA-232	64	16	64	1	>64	>128	1	>128	2	>128	0.5	>32	>128	>8	>16	>256	>32	>32
KM4	231	OXA-232	64	8	64	1	64	>128	1	>128	2	>128	0.5	>32	>128	>8	>16	>256	>32	>32
KM5	231	OXA-232	64	16	64	1	64	>128	1	>128	1	>128	1	>32	>128	>8	>16	>256	>32	>32
KM6	231	OXA-232	128	16	64	1	>64	>128	1	>128	2	>128	0.5	>32	>128	>8	>16	>256	>32	>32
KM7	231	OXA-232	>128	32	64	2	64	>128	1	>128	1	>128	1	>32	>128	>8	>16	>256	>32	>32
KM8	231	OXA-232	64	16	64	1	64	128	0.5	>128	1	>128	0.5	>32	>128	>8	>16	>256	>32	>32
16-419	15	OXA-232	>128	32	>64	4	64	>128	2	>128	2	>128	0.5	>32	>128	>8	>16	>256	>32	>32
16-422	15	OXA-232	128	32	64	2	32	128	0.5	>128	2	>128	0.5	>32	>128	>8	>16	>256	>32	>32
16-423	15	OXA-232	32	2	16	1	64	>128	0.5	>128	0.25	>128	0.5	>32	>128	>8	>16	>256	>32	>32
16-424	15	OXA-232	2	4	16	1	64	>128	0.5	>128	0.25	>128	0.5	>32	>128	>8	>16	>256	>32	>32
16-425	15	OXA-232	32	2	16	1	64	>128	0.5	>128	0.25	>128	0.5	>32	>128	>8	>16	>256	>32	>32
16-426	15	OXA-232	>32	>16	>16	1	64	>128	4	>32	0.25	>128	0.5	>32	>128	>8	>16	>256	>32	>32
16-427	15	OXA-232	128	32	64	1	32	64	0.5	>128	2	>128	0.5	>32	>128	>8	>16	>256	>32	>32
16-457	15	OXA-232	128	32	64	2	32	128	0.5	>128	2	>128	0.25	>32	>128	>8	>16	>256	>32	>32
16-458	15	OXA-232	128	32	64	2	32	128	0.5	>128	0.25	>128	0.25	>32	>128	>8	>16	>256	>32	>32
16-459	15	OXA-232	32	2	8	1	64	>128	0.5	>128	0.25	>128	0.25	>32	>128	>8	>16	>256	>32	>32
16-460	15	OXA-232	32	2	16	1	64	>128	0.5	>128	0.25	>128	0.25	>32	>128	>8	>16	>256	>32	>32
16-462	15	OXA-232	128	32	64	2	>64	>128	0.5	>128	0.25	>128	0.25	>32	>128	>8	>16	>256	>32	>32
ET1	15	OXA-232	16	0.5	4	1	32	128	1	>128	2	>128	0.5	>32	>128	>8	>16	>256	>32	>32
ET2	15	OXA-232	128	16	64	1	64	128	0.5	>128	4	>128	1	>32	>128	>8	>16	>256	>32	>32
ET3	15	OXA-232	128	32	64	2	64	128	1	>128	2	>128	0.25	>32	>128	>8	>16	>256	>32	>32
ET4	15	OXA-232	128	32	64	1	64	128	0.5	>128	2	>128	0.25	>32	>128	>8	>16	>256	>32	>32
ET5	15	OXA-232	128	16	64	1	32	128	0.5	>128	2	>128	0.25	>32	>128	>8	>16	>256	>32	>32
ET6	15	OXA-232	16	2	4	0.5	16	>128	0.25	>128	2	>128	0.5	>32	>128	>8	>16	>256	>32	>32
ET7	15	OXA-232	128	8	32	0.5	32	128	0.5	>128	2	>128	0.25	>32	>128	>8	>16	>256	>32	>32
ET8	15	OXA-232	128	16	64	1	64	>128	1	>128	0.25	>128	0.25	>32	>128	>8	>16	>256	>32	>32
ET9	15	OXA-232	128	32	64	1	64	>128	0.5	>128	0.5	>128	0.25	>32	>128	>8	>16	>256	>32	>32
ET10	15	OXA-232	8	0.5	8	1	64	128	0.5	128	0.25	>128	0.25	>32	>128	>8	>16	>256	>32	>32
ET11	15	OXA-232	128	16	64	1	64	>128	1	>128	0.5	>128	0.25	>32	>128	>8	>16	>256	>32	>32
ET12	15	OXA-232	16	4	16	0.25	32	128	0.5	128	0.25	>128	0.5	>32	>128	>8	>16	>256	>32	>32
ET13	15	OXA-232	16	0.5	4	0.5	16	128	0.5	128	2	>128	0.5	>32	>128	>8	>16	>256	>32	>32
ET14	15	OXA-232	128	32	64	1	64	128	0.5	>128	2	>128	0.25	>32	>128	>8	>16	>256	>32	>32
ET15	15	OXA-232	64	16	64	4	>64	>128	1	>128	0.25	>128	0.25	>32	>128	>8	>16	>256	>32	>32
ET16	15	OXA-232	32	2	32	2	>64	>128	0.5	>128	0.5	>128	0.25	>32	>128	>8	>16	>256	>32	>32
ET17	15	OXA-232	8	0.5	4	0.25	8	128	0.5	>128	0.5	>128	0.5	>32	>128	>8	>16	>256	>32	>32
ET20	15	OXA-232	16	0.5	4	0.25	16	128	0.5	>128	0.5	>128	0.5	>32	>128	>8	>16	>256	>32	>32
ET24	15	OXA-232	64	2	16	2	64	>128	0.5	>128	0.5	>128	0.5	>32	>128	>8	>16	>256	>32	>32
ET25	15	OXA-232	8	0.5	2	0.5	32	128	0.5	>128	0.25	>128	0.25	>32	>128	>8	>16	>256	>32	>32
ET26	15	OXA-232	16	0.5	4	0.5	8	64	0.5	>128	2	>128	0.5	>32	>128	>8	>16	>256	>32	>32
ET27	15	OXA-232	8	0.5	4	0.25	8	>128	0.5	>128	0.5	>128	0.5	>32	>128	>8	>16	>256	>32	>32
ET28	15	OXA-232	16	1	64	2	64	128	0.5	>128	0.5	>128	0.5	>32	>128	>8	>16	>256	>32	>32
ET29	15	OXA-232	128	32	>64	1	32	>128	0.5	>128	2	>128	0.5	>32	128	>8	>16	>256	>32	>32
ET31	15	OXA-232	128	32	64	2	32	128	0.5	>128	2	>128	0.5	>32	>128	>8	>16	>256	>32	>32
ET32	15	OXA-232	128	32	64	1	64	128	0.5	>128	2	>128	0.5	>32	>128	>8	>16	>256	>32	>32
ET33	15	OXA-232	128	32	64	2	32	128	1	>128	2	>128	0.5	>32	>128	>8	>16	>256	>32	>32
ET34	15	OXA-232	128	16	64	4	64	>128	0.5	>128	0.25	>128	0.5	>32	>128	>8	>16	>256	>32	>32
ET35	15	OXA-232	32	2	16	1	64	>128	0.5	>128	0.25	>128	0.5	>32	>128	>8	>16	>256	>32	>32
ET36	15	OXA-232	32	2	32	1	>64	>128	0.5	>128	0.5	>128	0.5	>32	>128	>8	>16	>256	>32	>32
ET37	15	OXA-232	8	1	4	0.5	32	128	0.5	>128	0.5	>128	0.5	>32	>128	>8	>16	>256	>32	>32
ET38	15	OXA-232	32	2	16	1	64	>128	0.5	>128	0.5	>128	0.5	>32	>128	>8	>16	>256	>32	>32
ET39	15	OXA-232	128	32	>64	1	16	128	0.5	>128	2	>128	0.5	>32	>128	>8	>16	>256	>32	>32
ET40	15	OXA-232	>128	64	>64	2	>64	128	0.5	>128	0.5	>128	0.5	>32	>128	>8	>16	>256	>32	>32
ET41	15	OXA-232	>128	64	>64	4	64	128	0.5	>128	0.25	>128	0.5	>32	>128	>8	>16	>256	>32	>32
ET42	15	OXA-232	128	32	64	1	16	64	0.5	>128	2	>128	0.5	>32	>128	>8	>16	>256	>32	>32
ET43	15	OXA-232	>128	64	>64	2	>64	>128	0.5	>128	0.25	>128	0.25	>32	>128	>8	>16	>256	>32	>32
ET44	15	OXA-232	128	32	64	1	16	64	0.5	>128	2	>128	0.5	>32	>128	>8	>16	>256	>32	>32
ET45	15	OXA-232	128	32	64	1	32	128	0.5	>128	2	>128	0.25	>32	>128	>8	>16	>256	>32	>32
ET46	15	OXA-232	32	2	16	1	64	>128	0.5	>128	0.5	>128	0.5	>32	>128	>8	>16	>256	>32	>32
ET47	15	OXA-232	128	64	>64	2	>64	>128	0.5	>128	0.25	>128	0.5	>32	>128	>8	>16	>256	>32	>32
ET48	15	OXA-232	128	32	64	2	64	128	0.5	>128	2	>128	0.25	>32	>128	>8	>16	>256	>32	>32
ET49	15	OXA-232	128	32	64	1	16	64	1	>128	2	>128	0.5	>32	>128	>8	>16	>256	>32	>32
ET50	15	OXA-232	128	32	6	1	32	64	0.5	>128	2	>128	0.5	>32	>128	>8	>16	>256	>32	>32
ET51	15	OXA-232	16	2	8	0.5	16	128	0.5	>128	2	>128	0.5	>32	>128	>8	>16	>256	>32	>32
ET52	15	OXA-232	128	32	64	1	32	128	0.5	>128	2	>128	0.5	>32	>128	>8	>16	>256	>32	>32
ET53	15	OXA-232	128	32	64	0.5	32	128	1	>128	0.5	>128	0.5	>32	>128	>8	>16	>256	>32	>32
ET54	15	OXA-232	128	32	>64	1	64	128	0.5	>128	2	>128	0.5	>32	>128	>8	>16	>256	>32	>32
ET55	15	OXA-232	32	2	16	1	64	>128	0.5	>128	0.5	>128	0.25	>32	>128	>8	>16	>256	>32	>32
ET56	ST15-1LV	OXA-232	128	16	>16	0.5	32	128	1	>128	0.25	>128	0.25	>32	>128	>8	>16	>256	>32	>32
ET88	15	OXA-232	128	16	>16	1	32	>128	0.5	>128	0.25	>128	0.25	>32	>128	>8	>16	>256	>32	>32
EK32	15	OXA-232	16	1	4	0.5	16	64	0.25	>128	2	>128	0.5	>32	>128	>8	>16	>256	>32	>32
EK45	15	OXA-232	>128	32	64	0.5	16	64	0.25	>128	2	>128	0.5	>32	>128	>8	>16	>256	>32	>32
DCW	15	OXA-232	32	2	8	16	0.5	128	0.25	128	2	>128	0.5	>32	>128	>8	>16	>256	>32	>32

a ETP, ertapenem; IPM, imipenem; MEM, meropenem; FPZ, cefepime-zidebactam; FPT, cefepime-tazobactam; CZT, ceftolozane-tazobactam; CZA, ceftazidime-avibactam; FEP, cefepime; TGC, tigecycline; AMK, amikacin; POL, polymyxin B; CAZ, ceftazidime; ATM, aztreonam; CIP, ciprofloxacin; LEV, levofloxacin; TZP, piperacillin-tazobactam; SXT, trimethoprim-sulfamethoxazole; CRO, ceftriaxone.

### Resistance gene profiles.

Whole-genome sequencing (WGS) was used to determine the profile of genes conferring resistance to antimicrobial agents (Table 3). Of all these isolates, 81 (100%) carried *bla*_OXA-232_, and no isolates carried other carbapenemase genes. All isolates carried the ESBL genes *bla*_CTX-M-15_ and *bla*_TEM-1B_ (100%), and *bla*_SHV-1_ was also identified in these isolates, including 14 *bla*_SHV-1_ (17.3%) and 67 *bla*_SHV-28_ (82.7%), while only 1 isolate carried a chromosomal class C beta-lactamase (AmpC) gene, *bla*_ACT-17_. No plasmid-carried AmpC genes were detected. All isolates carried the acquired 16S rRNA methyltransferase gene *rmtF*, conferring high levels of resistance to aminoglycosides. The predominant genes encoding aminoglycoside-modifying enzymes were those encoding *N*-acetyltransferases, the most abundant being *aacA4'-17* (81, 100%), followed by *aac(3′)-IV* (5, 6.2%). The nucleotidyltransferase *aadA2* was detected in 14 of the isolates (17.3%). All isolates (81, 100%) carried the ADP-ribosylating transferase rifamycin resistance gene *arr-2*. Plasmid-mediated fluoroquinolone resistance *qnr*-like determinants were detected in all isolates tested (81, 100%), and these were *qnrB1* (67, 82.7%) and *qnrS1* (14, 17.3%). Fluoroquinolone resistance gene mutations of GyrA (GyrA-S83I, 17.3%; GyrA-S83F and GyrA-D87A, 82.7%) and ParC (ParC-S80I, 100%) were found in all tested isolates.

**TABLE 3:** Distribution of carbapenemase genes and acquired resistance genes and mutations in K. pneumoniae isolates from China

ST (*n*)	KL loci (*n*) (%)	O loci (*n*) (%)	Virulence (*n*) (%)	Plasmids (*n*) (%)	ESBL genes (*n*)	Mutation (*n*)
ST15 (66)	KL112 (66) (100)	O2v1(66) (100)	fyuA (66_(100), irp1 and irp2 (66) (100), ybtAEPQSTUX (66) (100)	ColKP3 (66) (100), IncFIB(pKPHS1) (66) (100), ColRNAI (66) (100), IncHI1B(pNDM-MAR) (66) (100), repB (66) (100), IncFIB(pQil) (63) (95.5), Col440I (61) (92.4), IncFII(K) (55) (83.3), IncHI2A (1) (1.5), IncHI2 (1) (1.5%)	*bla*_CTX-M-15_ (66) *bla*_TEM-1B_ (66), *bla*_SHV-1_ (66), *bla*_ACT-17_ (1)	OmpK35, mutation (66), GyrA-83F (66), GyrA-87A (66), ParC-80I (66)
ST231 (14)	KL51 (14) (100)	O2v2 (66) (100)	fyuA (14) (100), irp1 and irp2 (14) (100), ybtAEPQSTUX (14) (100), IucBCD (14) (100)	ColKP3 (14) (100), IncFIB(pQil) (14) (100), IncFII(K) (14) (100), IncFIA (14) (100), IncFII(pAMA1167-NDM-5) (14) (100)	*bla*_CTX-M-15_ (14), *bla*_TEM-1B_ (14), *bla*_SHV-28_ (14)	OmpK35, mutation (14), OmpK36, truncated (13), OmpK36, mutation (13), GyrA-83I (14), ParC-80I (14)
ST-V (1)	KL112 (1) (100)	O2v1 (66) (100%)	fyuA (1 (100), irp1 and irp2 (1) (100), ybtAEPQSTUX (1) (100)	ColKP3 (1) (100), IncFIB(pQil) (1) (100), IncFIB(pKPHS1) (1) (100), ColRNAI (1) (100), Col440I (1) (100), IncHI1B(pNDM-MAR) (1) (100), repB (1) (100)	*bla*_CTX-M-15_ (1), *bla*_TEM-1B_ (1), *bla*_SHV-1_ (1)	OmpK35, mutation (1), GyrA-83F (1), GyrA-87A (1), ParC-80I (1)

### Virulence genes.

In total, four virulence features documented in the virulence factor database were detected in this study, namely, the yersiniabactin receptor gene (*fyuA*), the siderophore genes (*irp1* and *irp2*), the yersiniabactin siderophore cluster (ybtAEPQSTUX), and the aerobactin siderophore biosynthesis protein (IucBCD). The distribution of these virulence genes varied between strains: *fyuA*, *irp1*, and *irp2* and ybtAEPQSTUX were present in all STs strains, while IucBCD was present only in ST231. In short, the ST231 isolates had the highest number of virulence operons (four). The detection of virulence factors helps to understand the pathogenesis of different strains.

### Clonal distribution and plasmid repertoire.

**FIG 1:**
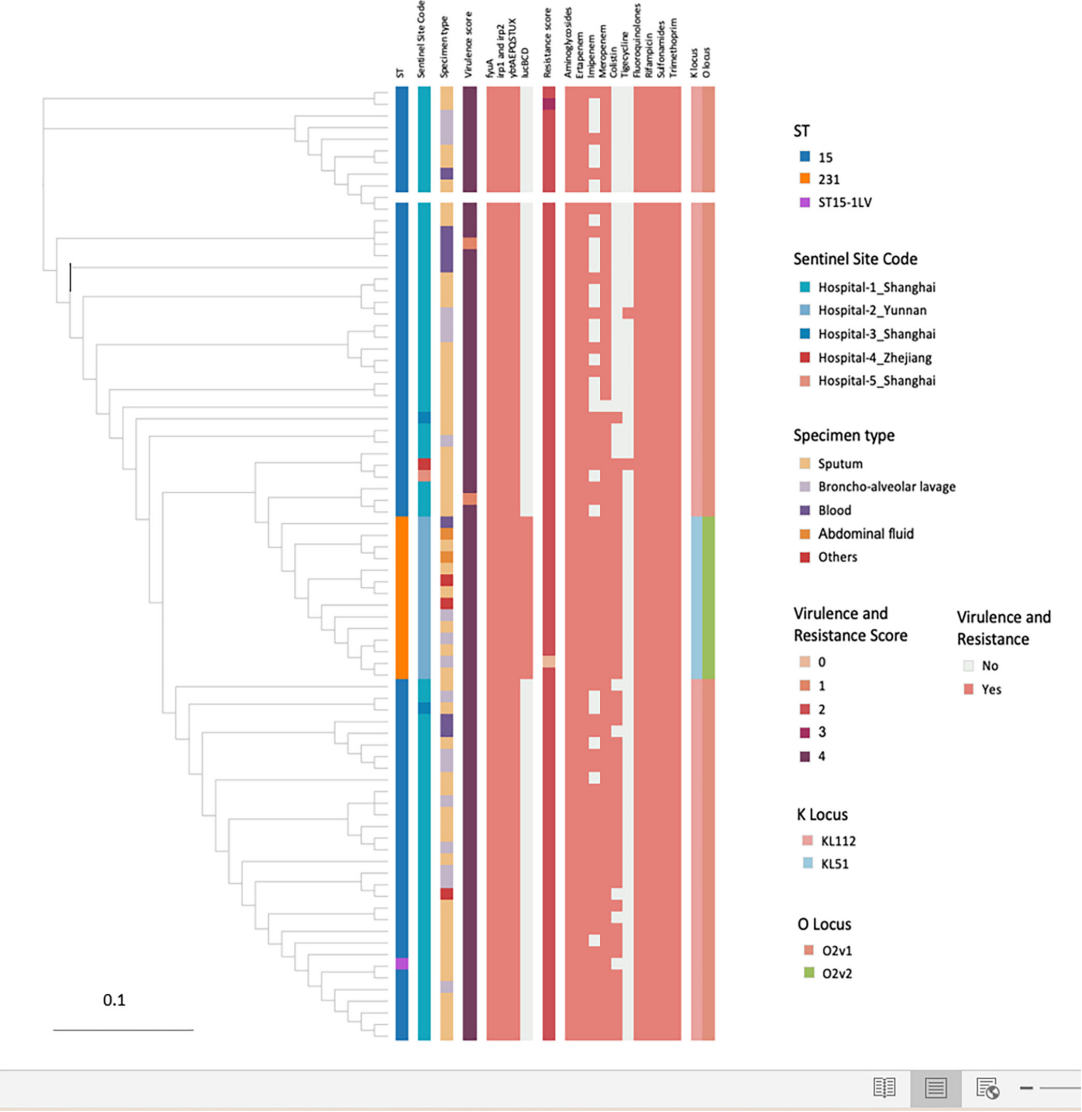
Phylogenetic analysis of isolates in this study. A phylogenetic tree of 81 genomes from China. The lineage highlighted in the box is shown in detail, with metadata blocks showing ST, K, and O loci, AMR, virulence factors, specimen type, and sentinel site code.

Overall, there were three sequence types (STs) among the K. pneumoniae isolates tested in this study, ST15 (*n* = 66), ST231 (*n* = 14), and ST-V (*n* = 1). The minimum-spanning tree indicated that the clonal transmission characteristics of the OXA-232-KP isolates are relatively dispersed. For example, clonal transmission of ST231 (*n* = 14) occurred in one hospital from Kunming, whereas ST15 occurred mainly in Shanghai and Zhejiang, which are geographically close to each other (Fig. 1). Two K-locus types, KL112 (67, 82.7%) and KL51 (14, 17.3%), were identified in this study. The correlation between capsular locus type and sequence type was also clarified by the minimum-spanning tree: KL51 with ST231 and KL112 with ST15. With regard to O antigen types, all isolates belonged to O1. The O locus types also showed an ST-specific distribution. In particular, O2v1 was found in ST15, whereas O2v2 was found in ST231. The predominant plasmid replicons present in the 81 K. pneumoniae isolates were ColKP3 (100%), IncFIB-like (100%), and IncFII (K) (85.2%). Other plasmid replicons detected were ColRNAI, repB, IncHI1B(pNDM-MAR), Col440I, IncFIA, IncHI2A, and IncHI2. In this study, we observed that both ST15 and ST231 isolates carried the ColKP3 plasmid replicon.

### Genetic context surrounding *bla*_OXA-232_ and *rmtF*.

**FIG 2:**
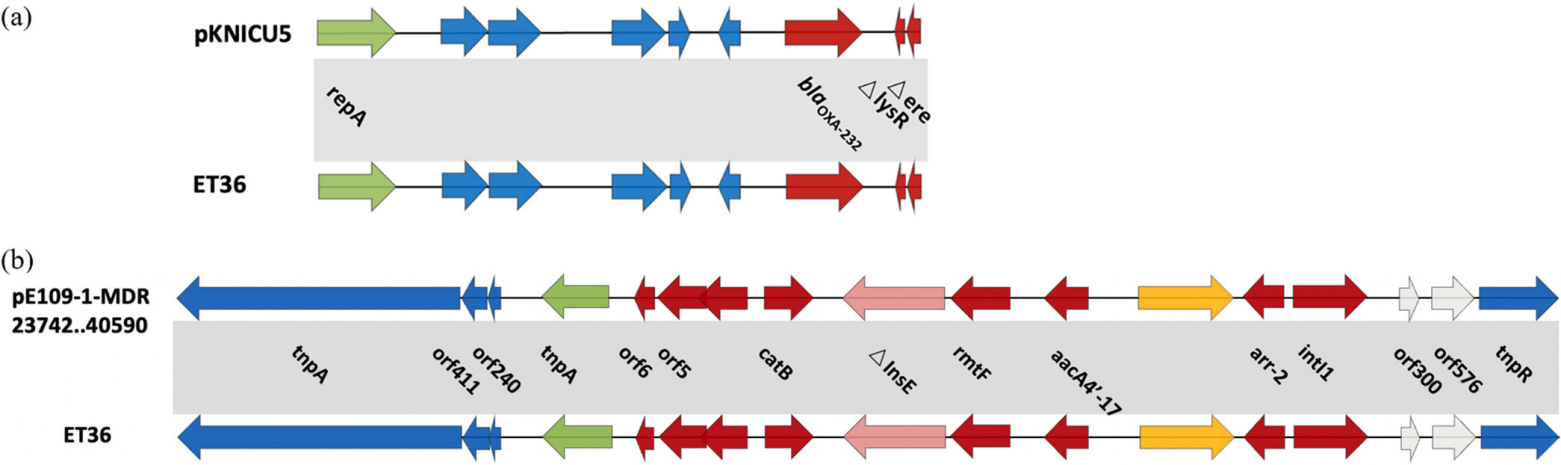
(a) Comparison of the genetic context of the OXA-232 gene in this study with the reference plasmid pKNICU5. (b) Comparison of the genetic context of the *rmtF* gene in this study with the partial fragment of the reference plasmid pE109-1-MD. Color genes, genetic elements, and other traits are shown according to functional classification. Shading indicates regions of homology (.99% nucleotide identity).

The 81 isolates in our study all shared an identical OXA-232-carrying genetic context that was located within the truncated Tn*2013*-like insertion sequence (IS)-based transposition, with the best identity to a ColKP3 plasmid, pKNICU5 (KY454616), of 6,141 bp (Fig. 2a). As for the 16S rRNA methyltransferase gene *rmtF*, which is not in the same plasmid with OXA-232, the analysis results showed that *rmtF* is located on the IncFIB-type plasmid and is in tandem with *arr-2*, *aacA4'-17*, and *catB* at transposon Tn*6867b*. BLAST results showed that the plasmid containing the *rmtF* gene had the highest identity (>99%) with pE109-1-MDR (CM012200) (Fig. 2b). The only difference was that there was a point mutation in the *rmtF* gene in strain ET39, causing an amino acid substitution (D249E), but the antimicrobial susceptibility of ET39 did not change significantly compared to other isolates. The introduction of some functional changes may require further investigation.

## DISCUSSION

Following the first description of an OXA-232-producing K. pneumoniae isolate detected in a tertiary hospital in Shanghai, China, which caused a small-scale clonal dissemination, the emergence of OXA-232-KP has been reported (22, 25–27). At present, OXA-232-producing K. pneumoniae isolates are usually isolated from vulnerable populations such as neonates and elderly patients exposed to medical facilities and have occurred extensively in neonatal intensive care units and intensive care units (25, 26, 28). To date, there have been no large-scale genomic studies of isolates carrying OXA-232 in China, which is of great importance in understanding their emergence and distribution in different geographical regions for public health initiatives (13). According to previous reports, most OXA-48-like carbapenemases hydrolyze carbapenems poorly, while some OXA-48-like variants (such as OXA-163, OXA-247, and OXA-405) hydrolyze only extended-spectrum cephalosporins but not carbapenems (14). However, in association with the impaired permeability or the production of extended-spectrum β-lactamases, some OXA-48-like enzymes can confer high levels of carbapenem resistance, similar to OXA-232 (14, 29).

Clonal lineages of K. pneumoniae differ in their ability to acquire resistance, virulence genes, and propensity to spread within hospital and community settings (30). The high prevalence of ST15 carbapenemase-producing *Enterobacteriaceae* (CPE) harboring *bla*_OXA-232_ was consistent with published data from China (14, 22, 31). There were two novel findings in this study. ST231 K. pneumoniae harboring *bla*_OXA-232_ was reported for the first time in China, and others have been reported in India, Switzerland, and Brunei (9, 12, 32). In addition, the novel ST (ST-V, gapA1-infB1-mdh-1-pgi8-phoE1-rpoB1-tonB447) was a single-locus variant of ST15 with variation at the *tonB* gene. KL51 was reported from the United States, Sweden, the United Kingdom (33), Thailand (34), and Lebanon (35). KL112 has been reported from Russia (36). Understanding such vectors carrying the resistance genes could help improve strategies to control the spread of AMR (37). The association between the ColKP3 replicon and the *bla*_OXA-232_ gene has also been observed in ST231 or ST15 isolates around the world (37, 38). To the best of our knowledge, plasmid pKNICU5 was isolated from an ST15 OXA-232-producing K. pneumoniae isolate in a children’s hospital in Shanghai in 2017, suggesting that the plasmid carrying OXA-232 has been widely disseminated in China in recent years (22).

### Conclusions.

Our findings showed that OXA-232-producing K. pneumoniae has increased and spread to different geographical areas of China through interhospital and interregional dissemination of a few high-risk K. pneumoniae clones (ST15, ST231, and ST-V). The spread of *bla*_OXA_-like genes in China was due to the spread of the plasmid groups ColKP3, IncFIB, and IncFII (K).

## MATERIALS AND METHODS

### Strain collection and antimicrobial susceptibility testing.

**FIG 3:**
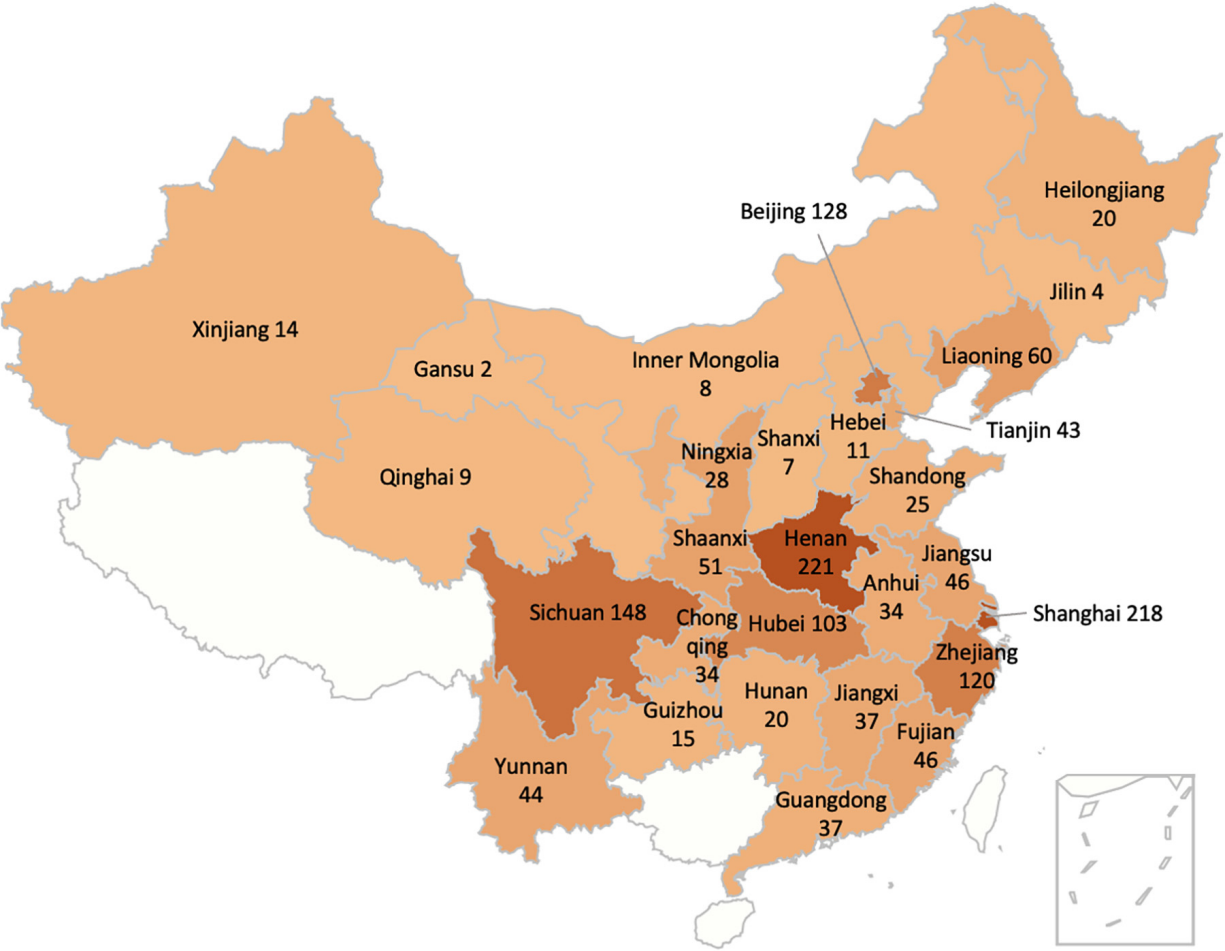
Geographic distribution of the 1,533 CRKP isolates collected from 2016 to 2021.

Of the 1,533 carbapenem-resistant K. pneumoniae (CRKP) isolates collected from 2016 to 2021 by the China Antimicrobial Surveillance Network (www.chinets.com, 51 medical centers in 28 Chinese provinces or cities), 81 nonduplicate strains carrying the OXA-232 gene were screened (Fig. 3). All isolates were identified by matrix-assisted laser desorption ionization–time of flight mass spectrometry (MALDI-TOF MS; bioMérieux, France) and 16S rRNA gene sequencing. MICs were determined with the Clinical and Laboratory Standards Institute (CLSI) recommended reference broth microdilution method using CLSI M100 31 resistance breakpoints for ampicillin, piperacillin-tazobactam, cefazolin, ceftazidime, cefepime, imipenem, meropenem, aztreonam, levofloxacin, amikacin, ceftazidime-avibactam, and trimethoprim-sulfamethoxazole (39). Tigecycline MICs were interpreted using the U.S. Food and Drug Administration MIC breakpoints for *Enterobacterales* (40). Cefepime-zidebactam and cefepime-tazobactam MICs were interpreted using CLSI breakpoints for cefepime for comparative purposes only. European Committee on Antimicrobial Susceptibility Testing (41) (EUCAST) MIC interpretive breakpoints were used for polymyxin B.

### Whole-genome sequencing and sequence analysis.

DNA was extracted using a commercial midi kit (Qiagen, Hilden, Germany) according to the manufacture’s recommendations. Sequencing libraries were prepared using the Nextera XT DNA library preparation protocol (Illumina, San Diego, CA, USA), and then the genomic DNA was subjected to Illumina short-read sequencing (150-bp paired-end reads). Reads were trimmed using Sickle (GitHub). Draft genome assemblies were generated using SPAdes 3.12.0. Further assembly was performed by mapping contigs on the reference plasmid sequences (>99% identical), and the assembly was confirmed using the PCR-based gap closure method. Antimicrobial resistance genes as well as multilocus sequence typing (MLST), mobile elements, outer membrane protein-coding genes (OmpK35, OmpK36), resistance-related mutants, and other features were detected using the following databases: Resfinder (42), BIGSdb-Pasteur MLST database (43), ISfinder (44), CARD (45), and BIGSdb (46). Genome annotation was performed using RAST (47) and BLASTp/BLASTn (48) searches for open reading frames (ORFs) and pseudogenes. BRIG (BLAST Ring Image Generator) software was used to compare genetic context sequences (49).

Kleborate software was used to analyze K and O antigen types, virulence genes, and the integrative conjugative element (ICEKp) (50). The four major virulence loci were screened, including the siderophores yersiniabactin (*ybt*), aerobactin (*iuc*), and salmochelin (*iro*), the genotoxin colibactin (*clb*), and ICEKp, a virulence-associated mobile genetic element (MGE) of K. pneumoniae (51). The plasmid types in the CRE isolates were identified using SRST2 v0.2.0 with the PlasmidFinder (44) database from the Centre for Genomic Epidemiology, v2021–01-13. A maximum-likelihood phylogenetic tree was reconstructed from SNPs across the genome using Snippy v4.4.3 (https://github.com/tseemann/snippy) with default parameters (minimum read mapping quality to consider, 60; minimum base quality to consider, 13; minimum site depth for calling alleles, 10; minimum proportion for variant evidence, auto; minimum quality in variant call format (VCF) column 6, 100; minimum soft clipping to allow, 10). The phylogenetic tree was visualized and beautified using FigTree v1.4.2 (http://tree.bio.ed.ac.uk/software/).

### Ethical approval.

The study protocol was approved by the Institutional Review Board of Huashan Hospital, Fudan University (number 2018-408).

### Data availability.

Sequence data were submitted to the National Centre for Biotechnology Information (PRJNA819578).
